# Medical Miscellanies

**Published:** 1818-01

**Authors:** 


### PART IV.

### MEDICAL MISCELLANIES.

### THE PORTE FEUILLE, No. X.

OR,

DEPOT FOR DETACHED FACTS AND INTERESTING PHÆNOMENA, OCCURRING IN THE PRACTICE OF THE FRIENDS OF THE MEDICO-CHIRURGICAL JOURNAL.

"——Trahit quodcunque potest
Atque addit acervo——"

*Punctured Wound of the Eye.*

William Webb, 20th March, 1809, while employed in a boat alongside a ship, had the pike of a boat-hook struck immediately through the pupil of the left eye, through which opening were evacuated all the humours at once. A compress of linen was immediately applied over it, secured by a bandage, and kept constantly wet with the refrigerant lotion. Forty ounces of blood were extracted, an opiate exhibited, and he was put to bed.—29th. Slept well; in no pain; a cathartic was prescribed. Between this period and the 13th of April, the wound healed without any pain, tumefaction, or inflammation of the parts whatever, and he was discharged perfectly cured. C.

---

*Severe Pneumonia.*

A marine, aged 26, belonging to a ship in Hamoaze, Feb. 7th, 1809, had, a few days before, complained of a slight cold, for which he took evacuants: he this day returned with every symptom of severe pneumonia; he was immediately bled to the amount of forty ounces; took an emetic of antim. tart.; after the operation of which, a brisk cathartic; and a diaphoretic in the evening.—8th. Considerably better; cough troublesome. Cont. diaphoretic.—9th. Slept badly the preceding night; omnia symptomata exacerbata. V. S. ad ℥xl; to take a powder of calomel and jalap three times a day.—10th. Felt relieved by the bleeding and cathartic, which operated copiously. Cough continues troublesome, and the oppression of breathing is very great. Rep. V. S. ad ℥xxxvj; blisters to the back and chest, accompanied with diaphoretics.—11th. Spent a restless night; febrile symptoms severe. Rep. pulv. cathart. ut 9no. and apply blisters to the sides.—12th. He has no pain, and thinks himself better; pulse, heat, and thirst, however, indicate much danger. Rep. cath. ut heri. Was this afternoon sent to the hospital, where he died soon after. This appears to be one of those unhappy cases where the parenchymatous substance of the lungs was so morbidly affected, that no medical means, perhaps, would have saved life. Or did effusion take place?

NAUTA.

---

*Necrosis of the Tibia in a Boy, with Amputation.*

A boy, of rather a scrofulous appearance, became affected with a series of abscesses in the left thigh, when about seven years of age. These abscesses descended to the leg, some of those in the thigh healing slowly, and leaving a puckered state of the integuments. At length the leg swelled enormously, and numerous abscesses discharged matter of a fœtor expressive of diseased bone. The poor boy became bed-ridden and neglected; maggots poured forth in abundance, from various openings, throughout the summer of 1816, he being then in his ninth year. In the summer of 1817, the abscesses in the thigh were nearly healed; the knee-joint appeared distorted; the leg and foot of an amazing size, and grown in such positions as to be for ever useless. Dr. Johnson proposed amputation of the thigh; and the boy, eight years and seven months old, joyfully agreed, and crawled, himself, into the room where the operation was to be performed, with the most tranquil countenance.

The first, and every subsequent incision, disclosed nothing but a substance perfectly resembling *brawn*, and without a single trace of muscular fibre. The bone being sawed, the blood-vessels were seen preserving their natural calibre, like some of the vessels in the cut surfaces of the liver. They could not be drawn out, and were with difficulty tied. Almost every one prognosticated a bad result: but the flaps adhered and remained so, by the *first intention;* and the boy was soon hopping about.

The knee-joint was completely dislocated; the condyles of the femur were growing to the inner side of the tibia, and the patella was amalgamated into the anchylosed joint: the whole tibia, from one epiphysis to another, was dead, and a great part of it dissolved and gone. A new case was nearly complete, excepting a number of holes through which the old bone was draining in solution. Dr. Johnson was making a preparation of it; but a dog dragged it out of a bucket of water, where it was macerating in the military hospital, and demolished it completely. J. J.

---

*Clinical Cases in Public Hospitals.* HEMIPLEGIA.

Mary-Anne Chapel, ætat. 16, was admitted into Guy's hospital this day [January 22, 1817], speechless; and only the following particulars could be learnt. She has been ill about fourteen days; was perfectly well before the first attack, which occurred a fortnight ago. Menses have never appeared. The *right* side is hemiplegic; cannot articulate; retains the urine and fæces, and appears much distressed by a fixed pain in the *left* side; pulse 100; general health not much impaired; has had two distinct attacks, since the last of which she has remained in the state above mentioned. *Purgative.*—23d Jan. She took two doses of scammony and calomel, which gave her four motions. She is now sensible, and, by signs, informs us, that the pain in the head is gone. Slept some last night. Pulse 84; countenance somewhat flushed. *Four leeches to the left temple; blister to the shaven head. Infus. rosæ with sulph. sodæ ter dic.*— 24th Jan. Some slight degree of sensibility and motive power in the paralyzed leg, but not in the arm. Makes attempts at articulation. Bowels costive; leeches have bled freely; blister not yet risen. Pil. colocynth. c. calomelan. gr. x statim.—25th. Seven loose motions from the pills. Slept some hours; blister risen well. *Rep. magnes. sulphat.*—26th. Costive. *Rep. pil. purgant. gr. viij.*—27th. The mobility of the paralytic leg so much recovered that she put it to the ground. Arm still motionless; bowels open.—29th. Has no vertigo or pain any where; bears the erect posture well; head drawn to the left side; bowels costive. *Pil. hyd. gr. iv. omni nocte; pil. colocynth. c. cal. gr. viij; a seton in the neck.* Camphor julep ter die.—3d Feb. Greater ability to articulate; other symptoms stationary.—5th. Has almost entirely recovered the power of speech; can walk with very little assistance; no return of power in the affected arm, but has some sensation there. Pulse natural; bowels open. *Contin. pil. hyd.*—6th. To take two grains of the *nux vomica* three times a day in the form of pill.—8th. Stated to-day, that she had been subject to pain in the left side of the head for about six months prior to the attack. *Contin. nux vomica et pil. hyd.*—10th. Has considerable power in the right leg; speaks without difficulty; sensation, but not motion, of the arm returned. Increased the dose of nux vomica. She was gradually recovering, when the Reporter lost sight of her. S****.

(These Cases to be continued.)

---

*Case of severe pulmonic Disease, in which the Use of Digitalis was carried to a great Extent with Success.*

M. Corfinet (Pilot), a Frenchman of correct habits and delicate constitution, subject to catarrhal complaints, had, about fourteen months since, been confined nearly five months with (as he termed it) a severe cold. On the 14th of September, 1808, having for some time observed him cough severely, I asked him why he did not seek relief? His answer was, that he thought it would wear off as before. At this time the pulmonic symptoms were severe, especially the pain in the right breast, with puriform expectoration. On the preceding day, the primæ viæ were cleared, and to-day I prescribed the following draught: ℞. Tinct. digitalis gutt. x; tinct. scillæ ♏. xx; tinct. opii ♏. xx; aquæ hordei ℥ij; ft. haustus bis die. His diet (from choice) consists chiefly of vegetables, puddings, &c. — 15th, 16th, 17th, 18th, 19th, 20th. The dose of digitalis is now 60 drops, being increased five drops each day. The squills have been omitted since the 17th, but the tinct. opii is continued; and the medicines appear to agree perfectly. — 21st. No remarkable effects from the digitalis; pulse varies from 80 to 100; the cough is decidedly better, and the expectoration lessened, *Cont. medic. ut heri.* — 22d. Medicine made him sick; reduce the digitalis to 50 drops. — 23d. Continues to improve; 60 drops bis die.—24th. Experienced considerable sickness from the digitalis, which is to be lessened to 30 drops, with gr. x of pulv. ipec. c. bis die; tinct. opii ut antea.—25th. He is better. T. digitalis gutt. xx ter die. *Cont. alia medicam.* — 26th. Thirty drops ter die. — 27th. The sedative powers of the digitalis have at length affected the pulse and pupils, but produce no sickness at stomach. Cough and expectoration almost gone, and the latter has lost its purulent appearance. Increase the digitalis to gutt. xxxv ter die. — 28th. To take 40 drops of T. digital. and laudanum three times a day; omit the Dover's powder. — 29th. The effects of the digitalis are now very evident. Between this period and the 4th of October, the dose was increased to 120 drops three times a day, when the tinct. opii was omitted. — 5th. The medicine did not agree so well as usual, owing to the omission of the laudanum, but he continues to improve rapidly. Continued the use of the digitalis, increasing the quantity each day, till the 10th, when in the course of the day he took 540 drops of tinctura digitalis. The effects of the medicine being rather violent, I consider it as pushed to its farthest extent. Pulmonic symptoms are now completely subdued; and as he is apprehensive of a return, if he continues at sea, he was this day (11th Oct.) sent on shore.—J. CUNNINGHAM, *Surg. R. N.*

### MISCELLANIES.

Quicquid agunt Homines.

### *On the Ætiology of Tropical Fever.* By Dr. Dickson, F. R. S. E. and L. S. Physician to the Clifton Dispensary.*

In the approbation which, in your last Number, you have bestowed on Dr. Fergusson's "Inquiry into the Origin and Nature of the Yellow Fever," I cordially join; but to that part which appears, somewhat at his expence, to convey a compliment to myself and others, I cannot accede; for I am of opinion, that by additional facts and judicious observations, he has contributed materially farther to illustrate the origin and *habitat* of the Tropical Endemic; and, with reference to myself (allowing for the time requisite to transmit communications to and from the West Indies) that he could not have seen the Paper to which you more particularly allude; as it appeared in the Edinburgh Medical and Surgical Journal for January 1817, and his Communication was read before the Medical and Chirurgical Society, on the 18th of March following.

With respect to the fever in the Childers, I perfectly agree with you, that it had the same origin as that which occurred in the Dart in 1807; and which I have witnessed in a variety of other instances from the same cause, a foul state of the hold, in the West Indies; and I observe, that Dr. Fergusson, with much candour, has since adopted this conclusion by the following quotation from his subsequent communications, which appear in Dr. Bancroft's recent publication; and which you had not seen at the time of writing your remarks. After having detailed, in a previous communication, the sickly condition in which the Childers reached Carlisle Bay, and her having been afterwards blown from that anchorage, Dr. Fergusson proceeds: "At the close of the last Report, we left the Childers sloop of war under circumstances of peculiar uncertainty, and distress. She, however, arrived safe at Antigua, where the purifications intended for her at Barbadoes were promptly ordered at the dock-yard, (English Harbour), when the hold of the ship was found to be in so extraordinary a state of filth, fermentation and impurity, as most amply to account, in the opinion of the Medical Staff of the Navy, for the rise, progress, and continuance of the fever that prevailed on board her. *Since* her purification (clearing the hold), she has continued perfectly healthy, though she has taken many fresh hands on board." P. 230-1.

* We are sorry that the Original Department was so far set up when Dr. D's Paper arrived, that we were obliged to place it in this Department. Edit.

In addition to these Reports, Dr. Fergusson says, in a letter to Dr. Bancroft, "I had two documents respecting the *foul* state of the *hold* of the Childers, which together make a very strong case indeed:" the one was from Dr. Wray, Physician to the Forces, the other from Mr. Neill, the intelligent Surgeon of the Flag Ship, amply establishing the very noxious and offensive effluvia from that vessel's hold. *Bancroft's Sequel,* 232 *et seq.*

Relative to the very unhealthy situation of the town of Fort Royal, and of Fort Edward, Martinique, I can fully corroborate the remarks of Dr. Fergusson in the last volume of the Medico-Chirurgical Transactions, p. 119 et seq. After the capture of the island in 1809, we re-occupied the building in Fort Royal, which had formerly been the Naval Hospital; and I might shew from various representations of my own to the Commander in Chief, and to the Naval Medical Board, the extreme unhealthiness of its situation, on which account orders were issued that men should be sent there as seldom as possible; and, finally, for its being totally discontinued; but I shall in preference subjoin the following Extracts from Reports made to me by Mr. Neil Smith, the then very able and zealous surgeon of that Institution.

On the 17th of November 1810, after noticing different improvements, he adds, "But I cannot alter the miasmata surrounding the Hospital. There is not a single soul belonging to the Establishment who has not been attacked with ague, except myself; and I do assure you that I live in *fear*, and I believe I may soon add, *trembling*. However, I have the consolation to know, that I have it in my power to arrest the fits in a very short time: the solution of arsenic is as sure, and, if cautiously given, as safe as any article in the Materia Medica, and I know none that possesses its powers in the disease alluded to." Mr. Smith's auto-prognosis was soon amply verified: in a subsequent Report, he observes "The fever column includes continued, remittent, and intermittent fever. Of the latter, very few were original; but whatever the type might be at the time of admission, it was sure to terminate in one or both of the latter forms." Again, on the 7th of February 1811, "I must here remark, that every patient that has lately been admitted, has been attacked with remittent or intermittent fever, in one form or another; and though the grounds are much clearer of woods, &c. than at any period heretofore, that does not lessen the prevalence of these complaints: there is something radically bad in this place, which no care or assiduity can controul."

He then alludes, in no very lenient terms, to the worse than inefficaciousness of bark; and in retaining his high opinion of arsenic, admits that neither these nor any other remedy will prevent the recurrence of the disease while exposed to its malarious influence. Mr. Mortimer, the predecessor of Mr. Smith, has also borne ample testimony to the unwholesome situation of Fort Royal, in his Report on the Yellow Fever inserted in the previous Numbers of your ably conducted Journal. I might farther adduce, if it were not altogether unnecessary, many documents to shew the prevalence there of the concentrated endemic, or of recurrent forms of fever, according to the season of the year, or the respective degrees of habituation.

I will not here follow Dr. Fergusson's excellent illustrations of the *locale* of yellow fever, by reference to the topography of the different islands; his conclusions nearly accord with my own experience, and particularly at Guadaloupe, where I had much opportunity of remarking the influence of site and elevation, (taking always into account the degree of predisposition, or of assimilation) upon the type and character of fever. But I may be permitted to remark, that the similarity of our conclusions afford the strongest presumption of their stability; seeing that they were formed by persons unacquainted with each other; on the same ground, but at different periods of servitude, and independently of extensive personal observation, founded upon the various Reports made to them, as the heads of the Military and Naval Medical Departments in the West Indies.

In making these observations, *a l'improviso*, and which, therefore, I fear stand in need of apology, I have to acknowledge a farther inducement than mere coincidence of opinion; a feeling of obligation to Dr. Fergusson and the army medical staff, for their care and attention to the unfortunate victims of yellow fever belonging to the navy. Not only every one who has witnessed the ravages of this dreadful scourge, with a harassing intensity of interest, which has invaded not only his waking but his sleeping hours; but every one, who has the welfare of the naval service at heart, will readily understand and participate this feeling on reading the following sentence: "At Barbadoes our hospitals of late have been in a regular course of importation of the Yellow Fever from the navy, but not even inoculation has been able to produce the disease upon any member of the hospital corps, by whom I may truly say, that the sick have been received with *open arms;* for the anti-social doctrines of ideal contagions, are not preached amongst us here, to the prejudice of duty and of humanity." *Med. Chir. Tr. vol. viii. p.* 143.

*December,* 1817.

---

### *Remarks on the Fever usually denominated Typhus.*

"Precept upon precept,
Line upon Line."

Blood-letting in fever needs nothing to be said, at this time of day, to recommend it to the notice of the profession. Experience has taught its important and beneficial effects: its employment has been farther sanctioned by many writers of ancient times; and some of the most eminent in the modern medical world have, at different periods, very ably employed their pens on the subject.

Waving, therefore, any attempt at saying aught that is new on the general subject, as to the medicinal powers of blood-letting, I shall only briefly relate what led me to use it as recommended below, and what were its effects in my own practice; adding a few practical hints, which, I hope, will make a remedy, still very frightful to many, not only pleasanter, but much more effectually, and, I hope, more extensively useful.

In the month of October, 1816, typhus fever made its appearance in this quarter, and continued to prevail for a considerable time afterwards, varying in its degrees of severity according as the constitution was more or less susceptible of its attack. Being then resident in the neighbourhood, I had frequent solicitations to visit the labouring poor afflicted with this complaint.

The disease was usually ushered in with alternate heats and chills, universal lassitude and great prostration of strength, dejected anxious countenance; dull and frequently slightly suffused eyes; head-ache; nausea; slight cough, and oppression about the præcordia, with pain in the loins, and irregular bowels. Pulse quick, small, feeble, and oppressed, in some cases: in others, soft and full, but easily compressible. Skin dry and warm; tongue white and moist; thirst considerable; stools black and fœtid.

The weather about this period was rainy, with a moist, cold, unwholesome state of the atmosphere; which, I reasonably concluded, must have predisposed, in a chief degree, to this disease; at least, in the majority of cases, I could detect no other very probable cause: that is to say, the greater part that came under my inspection were tolerably well clothed, and generally as comfortably lodged as people in that situation of life usually are; and, upon the whole, enjoyed an immunity from filth and the other evils of humble life. The most striking difference I observed, where cleanliness, ventilation, a due supply of clothing, and other necessaries were wanting, was, that the fever attacked whole families, instead of individuals of a family.

Being struck with the inertness of the practice adopted by the medical gentlemen in this quarter, and having had but too frequent opportunities of witnessing inflammatory fevers in southern latitudes; knowing, moreover, that such fevers, when neglected, or improperly treated in the first stage (that is, where evacuations had not been promptly and sufficiently used to subdue excitement), exhibited symptoms very similar to those usually met with in the typhoid fever of northern latitudes; and having learnt from experience, in such cases, that the general or local abstraction of blood from the system, aided by the use of mercury, either by itself or combined with opium and antimonial powder (according to existing circumstances), had the happiest effect in conducting the disease to a favourable issue, and tended much, in my opinion, to lessen the risk of subsequent disorganization and death, I determined to adopt a mode of treatment strictly analogous.

From such considerations, I was led to employ blood-letting, purgatives, with cold or tepid lavations, aided by calomel, in the first stage of this disease (typhus); and I am happy to state, that the result of such practice fully merited the confidence I had placed in it; as almost all the cases terminated favourably. Although the disease, in many instances, was not arrested, yet it passed through the different stages with symptoms comparatively mild, with little or no disturbance of the mental faculties, and evidently without any subsequent organic lesion.

The general or local excitement being thus overcome, or moderated, and the equilibrium of the circulation, in a certain degree, re-established, calomel was exhibited, not only to preserve a regular state of the bowels, but, by its specific action, to modify or change the diseased secretions and excretions. That it did so was evidently proved by inspection of the alvine evacuations, which at first were commonly thin, fœtid, and ill-conditioned; but gradually became of a light yellow colour, and changed from that to a dark brown on the specific effects of mercury becoming visible. The calomel was generally given at the commencement of the attack, in the proportion of a scruple for a dose, with the view of evacuating the bowels, which it seldom failed to effect in the course of eight or twelve hours after its exhibition, producing a few copious dejections, without much fatigue or irritation. Subsequently, it was administered as an alterative, in small doses, combined with opium.

In cases where I did not see the patients in the early stage of their illness, I frequently found the warm or tepid bath have the most salutary effects; namely, where there was a deficiency of heat on the surface of the body, or rather, where the heat was partial, it seemed to elicit the circulation to the extreme cuticular vessels, and in this manner unloaded the internal or visceral blood-vessels, and thus tended to restore a due equilibrium to the circulating system.

In cases where great collapse had taken place, the happiest results were experienced from small and repeated doses of warm Port wine, combined with aromatics, and occasionally a few drops of the tincture of opium. In such cases, I have also observed the best effects from the application of a large blister to the whole head and neck, allowing it to remain on for the space of twenty-four hours at least.

Although the state of the pulse and apparent debility in this disease did not, in many instances, indicate evacuations to be necessary; yet, whenever the head or chest were affected with an acute or dull pain, the sufferings of the patient were invariably mitigated by timely venæsection.

I am, therefore, decidedly of opinion, that at the commencement of this disease, the debility is only apparent, depending upon an increased action of the vessels of the brain or some other vital organ, producing (if I may so express myself), a suppressed action of the system. This, I think, may be fairly deduced from the first general or local subtraction of blood being often followed by such an increase of morbid heat, and of vascular action.

In submitting these hints to the public, I am aware of advancing nothing new or very interesting; yet I am anxious to call the attention of Practitioners to the utility of evacuations at the commencement of this disease. They have hitherto, I am persuaded, been too much neglected, or not carried to that extent necessary to insure success; either from the groundless apprehension of inducing debility, or from a fear of the insidious remarks and misrepresentations of the illiberal and unenlightened part of the profession.

*Nov. 7th*, 1817. B——.

### Note by the Editors.

We readily give insertion to the above remarks of our esteemed Correspondent, for two reasons; first, because from our personal knowledge of his talents, and the tone of his mind, we are sure that whatever he writes is the result of ample *real* experience, and unsophisticated observation:—in the second place, because we consider the remarks in themselves (though not absolutely original), to be valuable, and well calculated to promote *the good cause* all over the world.

We are among those who have long hoped to see a more energetic practice obtain in the treatment of the common fever of this country: we have contended that the term typhus should be regarded as a mere specific appellation, and that the fearful illusion which the very sound of the word occasions, should be dispelled. We have often wished that the celebrated hypotheses of spasm or debility should *now* atone for the ill "done in their days of nature," by being themselves "burnt and purged away:" and (as far as venæsection is concerned), have anxiously anticipated a restoration of the practice in fevers, to the good old method of Sydenham, Huxham, and Hippocrates.

This restoration, as might be expected, has gone on very slowly; though individuals here and there, for twenty years past, have employed blood-letting, both general and topical, in the fevers of Britain, still the practice has not been generally, nay, we believe, not even partially followed up till of late, by practitioners in civil life. We are credibly informed, that even in Edinburgh, that great fountain of medical knowledge, venæsection in fevers was never practised in the Royal Infirmary until within these nine months! The mighty improvement was first introduced, in the clinical wards, by that most able and meritorious physician Dr. Duncan, jun. The good effects were so apparent, that his example has been speedily and largely followed. This, indeed, is a triumph!

---

### *Short Extracts and Literary Intelligence.*

*Pemphigus.* A man, aged 70, was attacked, during convalescence from a simple mucous fever, with acute simultaneous pemphigus; and died on the eleventh day of the disease and sixth of the eruption. The latter had been accompanied at first with high fever, burning heat of the skin, and inflammation of the throat; and afterwards, delirium and a violent burning pain over the whole body. On the tenth day, many of the pustules contained, instead of serum, a sanious, though tolerably thick, pus. Others, having lost their epidermis, displayed ulcerated surfaces, of a deep red colour, and exquisitely sensible to the slightest touch. The tongue was dry, brownish in the centre; and the pulse hard, quick, and small. On the evening preceding death, the man had two brown and very offensive stools. They increased in frequency the following day. The pharynx, velum palati, and tonsils, were found, on dissection, evidently inflamed; and two vesicles, as large as a bean, occupied the inferior part of the pharynx.—*Recueil periodique de la Soc. de Méd. de Paris*, Avril 1817.

*Aortic Aneurism.*—A man, aged thirty-five, fond of hunting, who had enjoyed perfect health, and never complained of palpitation of the heart, expired suddenly one day as he was reading a gazette. On dissection, the pericardium was found full of blood, which had issued from the rupture of an aneurismal sac, formed by the aorta at its egress from the heart.—*Bulletin de l'Athen. de Méd. de Paris.* Dec. 1816.

*Curious Effect attributed to Saffron.*—Among the English, who crossed from Calais to Dover with M. Cadet, in a late visisit to England, there was one gentleman who wore upon his stomach a small bag of *saffron.* On being asked the reason of this, he said that he did it to prevent sea-sickness; and that he had become acquainted with such beneficent property of the vegetable, in the following manner:—A mercantile man, who had invariably been incommoded with vomiting, during the voyages which he was frequently under the necessity of taking, one day purchased about a pound of saffron; and, with a view of evading the heavy duty imposed upon it, concealed the packet in his shirt. During this passage, he experienced no inconvenience from sickness, although the sea was rough. The good effect in question he ascribed to the saffron; and communicated his conjectures to several others, who have repeated the experiment with success. From that time the narrator has never gone on board a vessel without his saffron packet. *Journal de Pharmacie.* Juillet 1817.

*Spleenwort.* This plant, the *asplenium trichomanes* of botanists, is much recommended on the continent for the cure of affections of the urinary bladder. One man, aged upwards of sixty, is reported to have been completely cured of *catarrhus vesicæ* of six years' duration, in which period he had frequently voided considerable quantities of urate of lime, and mucus, in his urine, and become much emaciated) by drinking every morning before breakfast, for two months (another account states every morning and evening) two glasses of an *infusion of spleenwort*, made in the proportion of two drachms (one ounce in the other account) of the plant to one *pinte* of boiling water. The urine gradually became clear, and the pain declined under its employment. *Gazette de Santé* and *Journal de Pharmacie*, Mars 1817.

---

Dr. Shirley Palmer, of Tamworth, one of the Editors of this Journal, has it in contemplation to publish a Translation, from the German, of Kreysig's new and elaborate work on "*Diseases of the Heart*," interspersed with practical remarks, and illustrated by numerous references to the works of Senac, Portal, Parry, Corvisart, Scarpa, Allan Burns, Hodgson, Farre, and all the best writers on lesions of the heart and large vessels, so as to form a complete Monograph on these diseases, including a sketch of their literary history.

Dr. Clough, Physician-Accoucheur to the St. Mary-le-bonne General Dispensary, to the Endeavour Society, and Benevolent Institutions, will commence his second Winter Course of Lectures on the Science and Practice of Midwifery, &c. on Monday the 5th of January, at half-past ten in the morning.

Mr. Guthrie, Deputy Inspector of Military Hospitals, will commence his Spring Course of Lectures on Surgery, on Monday the 19th of January, at five minutes past eight in the evening, in the Waiting-Room of the Royal Westminster Infirmary for Diseases of the Eye, Mary-le-bone Street, Piccadilly. To be continued on Mondays, Wednesdays, and Fridays.

Mr. Taunton will commence his Winter Course of Lectures on Anatomy, Physiology, Pathology, and Surgery on Saturday January the 24th, at eight o'clock in the evening precisely; and be continued every Tuesday, Thursday, and Saturday, at the same hour.

---

Bibliography (List of important Works, on the Medical Sciences, published upon the Continent, in the Close of 1816 and Commencement of 1817).

Elemens de Pathologie générale; par A. F. Chomel, D. M. 8vo. pp. 570. Paris.

Mémoires sur les maladies chroniques, les evacuations sanguines et l'acupuncture; par L. V. J. Berlioz, D. M. 8vo. Paris.

Mémoires de la Société Médicale d'Emulation; huitième vol. en deux parties, avec le portrait de Bichat, et quatorze gravures en taille-douce. Paris.

Histoire médicale générale et particulière des maladies épidémiques, contagieuses et epizootiques, qui ont paru en Europe depuis les temps les plus reculés, et notamment depuis le 14e. siècle jusqu'à nos jours; par J. A. F. Ozanam, D.M. &c. tom. v. 8vo. (The first volume only has yet appeared.)

Principes de Therapeutique appliqués aux maladies internes; par J. B. Achard-Lavort, D. M. &c. 8vo. pp. 618. Paris.

Controverses médicales (sur les metastases laiteuses et la péritonite); par R. G. Gastellier. 8vo. pp. 102. Paris.

Manuel de Syphilixie, ou notice sur le virus, les effets, la contagion, le traitement, les préservatifs, et les erreurs populaires, de la maladie vénérienne; par M. L. Fournier, D.M. 8vo. pp. 112. Paris.

Mémoire sur l'Hydrencéphale, ou Céphalite interne hydrencephalique; par J. F. Coindet. 8vo. Paris.

Traité du Délire, appliqué à la médecine, à la morale, et à la legislation; par F. E. Fodéré. Deux vol. 8vo. pp. 1166. Paris.

Nouvelles Recherches sur les maladies de l'Esprit, précédées de considerations sur les difficultés de l'art de guérir; par André Matthey, D. M. &c. 8vo. Paris.

Nosologie naturelle, ou les Maladies du corps humain, distribuées par familles; par J. L. Alibert. Deux vol. 4to. Paris.

Nouveaux Elémens de Therapeutique et de matière medicale, &c.; par J. L. Alibert. Quatrième Edition. Deux vol. 8vo. Paris. (We may here mention, that the tenth fasciculus of this author's splendid work, *Description des Maladies de la Peau*, is about to appear).

Tableaux chimiques du règne animal, ou Aperçu des résultats de toutes les analyses faites jusqu'à ce jour sur les animaux, &c.; par Jean-Frédéric John. 4to. Paris. (Translated from the German by Robert.)

Elémens de Chimie médicale; par M. P. Orfila. 2 vol. 8vo. Paris.

Précis élémentaire des maladies réputées chirurgicales; par J. Delpech. 3 vol. 8vo. Paris.

Des Hémorrhoïdes, ou Traité analytique de toutes les affections hémorrhoïdales; par A. J. de Montègre. 8vo. Paris.

Précis Elémentaire de Physiologie; par F. Majendie. 2 vol. 8vo. Paris.

Cours théorique et pratique d'accouchmens, &c.; par J. Capuron, D.M. &c. Paris.

Séméiologie générale, ou Traité des signes et de leur valeur dans les maladies; par F. J. Double. Tome ii, 8vo. Paris.

De la Goutte et des maladies goutteuses; par J. Guilbert, D. M. 8vo. Paris.
Nouveaux Principes de Chirurgie, &c.; par F. V. Legouas, D. M. 8vo. Paris.
Mémoire sur l'usage de la noix vomique dans le traitement de la paralysie; par M. Fouquier, D. M. 8vo. Paris.
Recherches anatomiques sur les hernies de l'abdomen; par Jules Coquet, D. M. &c. 4to. avec 4 planches. Paris.
Examen de Pathologie, &c.; par J. H. Réveillé-Parise, D. M. 8vo. Paris.
Médecine de l'armée d'Espagne en 1808, —9, et —10; par L. J. M. Lixon, Ex-médecin des camps et armées, &c. 8vo. Paris.
Police judiciaire pharmaco-chimique, ou Traité des Alimens, &c.: par W. H. G. Remer, D. M. &c. 8vo. pp. 424. Paris. (Translated from the German by Bouillon Lagrange and Vogel.)
Dictionnaire des sciences médicales (from vol. xvii. to xx. inclusive).
Des Maladies de l'Uterus, &c.; P. M. Nauche. 8vo. Paris.
Ueber die Krankheiten des Uterus (On the Diseases of the Womb); Von C. Wenzel. Fol. pp. 196 (with twelve plates). Mayence.
Entwurf einer allgemeinen Pathologie (Sketch of general Pathology); Von J. C. Reil. 8vo. Halle.
Die Krankheiten des Herzens systematisch bearbeitet und durch eigne Beobachtungen erlautert (Diseases of the Heart, systematically arranged, and illustrated by Cases); Von D. Friedrich Ludwig Kreysig. Dritter Theit. 8vo. pp. 415. Berlin. (The first part of this elaborate work, pp. 392, was published at Berlin in 1814; the second, pp. 879, in 1815; and the last has recently appeared.)
Handbuch der Pathologie, &c. (A Manual of Pathology, &c.); Von —— Raiman. 8vo. Vienna.

We shall, in future, publish a more correct and extended list of foreign works upon medicine and the accessary sciences, every three or six months, according to circumstances; and, in the intervals, occasionally announce the appearance or intended publication of the more important books. Edit.

---

